# Plant selection for ethnobotanical uses on the Amalfi Coast (Southern Italy)

**DOI:** 10.1186/s13002-015-0038-y

**Published:** 2015-07-15

**Authors:** V. Savo, R. Joy, G. Caneva, W. C. McClatchey

**Affiliations:** Hakai Institute, Simon Fraser University, 8888 University Drive, Burnaby, BC V5A 1S6 Canada; Department of Science, University Roma Tre, Viale Marconi 446, 00146 Rome, Italy; Department of Statistics and Actuarial Science, Simon Fraser University, 8888 University Drive, Burnaby, BC V5A 1S6 Canada; Botanical Research Institute of Texas, 1700 N. University Drive, Fort Worth, TX 76107-3400 USA

**Keywords:** Ethnobotany, Medicinal flora, Regression analysis, Pearson’s residuals

## Abstract

**Background:**

Many ethnobotanical studies have investigated selection criteria for medicinal and non-medicinal plants. In this paper we test several statistical methods using different ethnobotanical datasets in order to 1) define to which extent the nature of the datasets can affect the interpretation of results; 2) determine if the selection for different plant uses is based on phylogeny, or other selection criteria.

**Methods:**

We considered three different ethnobotanical datasets: two datasets of medicinal plants and a dataset of non-medicinal plants (handicraft production, domestic and agro-pastoral practices) and two floras of the Amalfi Coast. We performed residual analysis from linear regression, the binomial test and the Bayesian approach for calculating under-used and over-used plant families within ethnobotanical datasets. Percentages of agreement were calculated to compare the results of the analyses. We also analyzed the relationship between plant selection and phylogeny, chorology, life form and habitat using the chi-square test. Pearson’s residuals for each of the significant chi-square analyses were examined for investigating alternative hypotheses of plant selection criteria.

**Results:**

The three statistical analysis methods differed within the same dataset, and between different datasets and floras, but with some similarities. In the two medicinal datasets, only Lamiaceae was identified in both floras as an over-used family by all three statistical methods. All statistical methods in one flora agreed that Malvaceae was over-used and Poaceae under-used, but this was not found to be consistent with results of the second flora in which one statistical result was non-significant. All other families had some discrepancy in significance across methods, or floras. Significant over- or under-use was observed in only a minority of cases. The chi-square analyses were significant for phylogeny, life form and habitat. Pearson’s residuals indicated a non-random selection of woody species for non-medicinal uses and an under-use of plants of temperate forests for medicinal uses.

**Conclusions:**

Our study showed that selection criteria for plant uses (including medicinal) are not always based on phylogeny. The comparison of different statistical methods (regression, binomial and Bayesian) under different conditions led to the conclusion that the most conservative results are obtained using regression analysis.

**Electronic supplementary material:**

The online version of this article (doi:10.1186/s13002-015-0038-y) contains supplementary material, which is available to authorized users.

## Background

Plants and humans are engaged in a dynamic relationship, where plants evolve creating biodiversity and humans develop strategies and solutions. In this relationship, plants evolve secondary metabolites to protect themselves from being “used” and people find ways to use these metabolites to their advantage. Several aspects of this relationship have puzzled researchers over the past decades, especially those regarding the reasons behind plant selection criteria used by different communities around the world. In the ethnobotanical framework, theories have been expressed to explain possible mechanisms behind this selection (e.g., [1, 2]) and then knowledge transmission [3–5].

One selection criterion that has been hypothesized is based on phylogeny. A non-random distribution of used medicinal plant species across families has been observed in several parts of the world (e.g., [6–10]). Plants within the same family, with close evolution ties, more likely share similar secondary compounds [11] which may have similar or equal medicinal properties (e.g., [12]) and this has been intuitively discovered by many traditional communities. Furthermore, plants that are evolutionarily closely related have generally more total uses than those that are evolutionarily isolated [13].

Besides theories that suggest selection criteria based on phylogeny, many others have been hypothesized. Several researchers have looked at possible alternative criteria used in selecting medicinal plants (e.g., [14–18]). These are for example based on taste and smell [12, 14, 17, 19–21] but also shape (e.g., [14]) or different/combined features (e.g., [16, 18, 22]). Another hypothesized criterion, which can apply to different plant uses, follows the ethnobotanical apparency theory, which states that plants that are more common and/or more available generally have a higher cultural importance than less apparent plants [1, 23, 24]. This selection criterion is based on the fact that some plants are more easily available or visible, not necessarily more useful [25].

Cultural factors might also be important in selecting plants [26–28]. Other researchers have hypothesized that the selection of medicinal plants could have been influenced by medical treatises of the past, especially in the Mediterranean area [3, 4, 29–32]. However, some selection criteria must have been involved before these plant uses were firstly reported in written documents.

Different methods have been developed to validate these theories. Several statistical analysis methods may be used to analyze ethnobotanical data to highlight whether some taxa are more extensively used than others in a certain flora under the hypothesis that selection criteria are based on phylogeny. In this paper we investigate the use of three different statistical methods proposed to test whether a specific family is over or under-used in a certain area: residual analysis from linear regression [15, 33], the binomial test [2], and a Bayesian approach [34, 35]. Other researchers used also an imprecise Dirichlet modeling [36]. Turi and Murch [37] tested the Native American Ethnobotanical Database [38] using four statistical methods (linear regression, Bayesian, binomial and imprecise Dirichlet modeling). Kindscher et al. [39] analyzed three statistical methods (linear regression, Bayesian, and binomial) using a smaller dataset, focusing only on Native American medicinal plants used within the state of Kansas. While these methods often give similar results, there are differences between them (see [37]) that have given rise to disagreements among researchers, and up to now, there has been no clear consensus about the use of one method over another. We perform these analyses considering three different sets of ethnobotanical data for the same area (the Amalfi Coast, Southern Italy). We analyze selection criteria for medicinal plants but we also test the methods using a set of non-medicinal plants (handicraft production, domestic and agro-pastoral practices). However, the aim of this paper is not to define which statistical method is the best one, but to test if discrepancies in datasets, sample size, data collection and floras of reference could affect results in a way that has as large or larger an effect as the selection of the method itself.

This paper has two main objectives:

1. To distinguish whether differences in the selected datasets and analytic methods affect results and might, therefore, lead to different interpretations of plant selection criteria.
2. Test if the selection for traditional medicinal or non-medicinal plants is based on phylogeny, or is based on other selection criteria (chorology, life form, or habitat). We want to approach the analysis with an alternative hypothesis about selection of plants for use, and not just rely on a blind statistical method.

### Research area

**Fig. 1:**
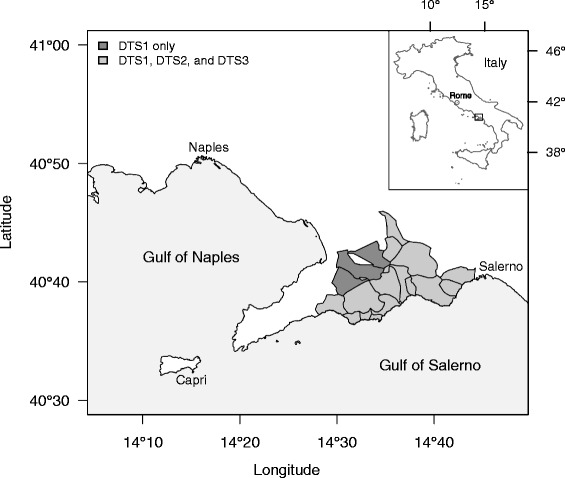
The Amalfi Coast (Italy) with boundaries of the research areas (for DTS1, DTS2 and DTS3)

The Amalfi Coast is located in Southern Italy (Campania) on the Southern side of the Sorrento Peninsula (Fig. 1). The area develops along the coast, but is typified by high mountains (up to 1400 m on the sea level) and steep slopes. These mountains belong to the Lattari chain, which constitutes an outcrop of carbonatic stratification, mainly composed by limestone, dolomitic limestone and Meso-Cenozoic dolomites [40].

The area has mainly a Mediterranean bioclimate (*sensu* [41, 42]), stretching into the Temperate bioclimate in the uplands. Rainfall in the area is rather high considering other Tyrrhenian coastal areas of Southern Italy (e.g., [43–45]). The average annual rainfall amount along the coast sums up to 1300–1400 mm, and is higher in some valleys (1800 mm) [46].

The geophysical diversity creates a multitude of habitats in close proximity, leading to a high plant and plant community diversity [47, 48]. This area has indeed attracted several botanists and other researchers over the centuries (e.g., [49–56]). Human impact has possibly contributed to increase this diversity creating a mosaic of woodlands, maquis, garrigues and pastures, according to the dissimilar utilization of plant communities. Indeed, the human presence in the area dates back to the Roman period [57] or even before. During the following centuries, local people ventured to the Mediterranean Sea for commercial activities, engaging relationships with different cultures. After the XI century, local people reduced their maritime activities and turned to agriculture, leading to an increase in cultivated land, especially on terraces [58]. These terraces are still part of the cultural landscape of the area, but agriculture in the area is declining as many terraces are being abandoned, while fewer are being cultivated with local crops and traditional techniques [59].

## Methods

Three different sets of data were analyzed in this paper:

- Dataset 1 (DTS1): Medicinal uses of plants in the 80-90ies (bibliographic source: [60, 61]).
- Dataset 2 (DTS2): Recent medicinal uses of plants (interviews between April 2007 and September 2009) (published data: [62]).
- Dataset 3 (DTS3): Uses of plants for handicraft production, domestic and agro-pastoral practices (interviews between April 2007 and September 2009) (original data).

The three datasets share similarities and differences in category of use, period of gathering and methodology of data collection and research area (Table 1, Fig. 1). Data on plant uses listed in DTS1 and DTS2 were obtained from literature [60–62]. However, data for DTS2 were collected by the first author of this paper with the same methodology and during the same period of collection of the data of DTS3. Data of DTS3 were collected during field surveys through 214 random semi-structured interviews with people who are or who have been living most of their lives in the area. Before starting interviews, informants were made aware of the scope of this study and Prior Informed Consent [63] was requested verbally. Interviews were conducted following the ISE Code of Ethics [64]. Personal data on the informants (age, job, place of residence) and on uses of local plants (vernacular name, place and period of gathering, how they use plants, part used, if they use fresh or dried plants, and if they use them with other plants, if the use is present or obsolete, etc.) were recorded. Plants quoted by informants were collected and voucher specimens were deposited at the *Herbarium* of the University Roma Tre (URT [65]). Plant species were identified following the “Flora d’Italia” [66] and their scientific names were updated [67–70].

**Table 1:** Differences and similarities in the three datasets (DTS1, DTS2, DTS3)

Characteristics	DTS1	DTS2	DTS3
Category of use	Same as DTS2	Same as DTS1	Different from DTS1 and DTS2
Period of data gathering	Different from DTS2 and DTS3	Same as DTS3	Same as DTS2
Methodology of data collection	Different from DTS2 and DTS3	Same as DTS3	Same as DTS2
Research area	Larger than that of DTS2 and DTS3 (includes the municipalities of Gragnano, Lettere, and Pimonte)	Same as DTS3	Same as DTS2

### The flora of the Amalfi Coast

**Fig. 2:**
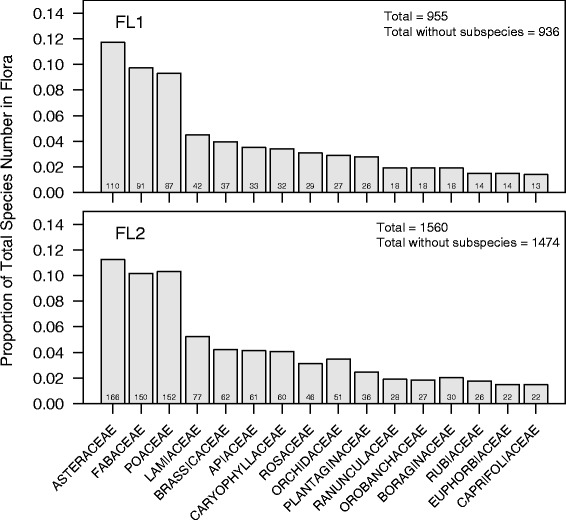
Proportion (and number) of species in the most represented families of the floras of the Amalfi Coast. The height of the bar represents the proportion of the flora contained in each of the top 16 families in FL1 (top panel), and the corresponding proportions of these same 16 families for FL2 appear in the bottom panel. The number of species in each family is written on the bar

The recent flora (FL1) [48] of the whole area includes 955 taxa (936 species- some species have more than one subspecies) belonging to 108 families (Fig. 2). However, since the datasets (DTS1, DTS2, DTS3) are related to different time periods and plants can become locally extinct, historical floras (e.g., [49–56]) were also considered. The whole set of data is defined here as the historical flora (FL2) of the Amalfi Coast. The historical flora (FL2) of the area includes 1560 taxa (1474 species) belonging to 116 families (Fig. 2). The majority of cultivated plants are also included in this flora. All plant species names (FL1 and FL2) were updated [69, 70], checking for possible errors [subspecies that are not recognized anymore, subspecies that are now considered different species, etc. (e.g., *Arenaria leptoclados* (Rchb.) Guss. and *Arenaria serpyllifolia* L. are now recognized as a unique species)]. Plant families follow APG III [71]. In this way all data were uniform as regards nomenclature and taxonomy.

The two floras (FL1, FL2) were characterized for their chorological and life spectra using information in Pignatti [66]) (Table 2). The percentages of specific life or chorological form vary (e.g., Phanerophytes constitute 13.5 % of FL1 and 11.9 % of FL2).

**Table 2:** Chorology, life form, habitat and taxonomical group (following [34,66]) of plants included in DTS1, DTS2, DTS3. Chorology, life form and taxonomical group distribution of FL1 and Fl2

Categories	DTS1	DTS2	DTS3	FL1	FL2
Species tot	176	102	45	955	1560
Taxonomical group					
Asterids	3	0	3	13	17
Campanulid/asterids2	29	18	3	173	274
Commelinids	5	2	3	108	199
Eudicots	6	1	0	25	44
Fabid/rosids1	11	3	1	39	61
Gymnosperms	4	0	0	5	15
Lamid/asterids1	46	23	7	162	256
Magnolids	1	1	1	3	5
Malvid/rosids2	29	22	5	156	252
Monocots	8	6	3	70	135
Pentapetalae	1	1	1	18	30
Pteridophytes	6	4	4	24	39
Rosids/n-fixing clade	27	21	14	158	234
Chorology					
Endemic	0	2	3	32	60
Circumboreal	12	2	2	33	60
Mediterranean	61	40	20	436	654
European	8	1	2	53	142
Eurasian	30	10	8	170	291
Asian and Australian	16	12	3	8	23
American	10	6	2	22	35
African	2	2	0	4	10
Paleotemperate	9	2	1	52	81
Wide distribution	28	15	3	136	173
Naturalized	0	0	0	1	2
Cultivated	2	2	0	0	7
Life form					
P (phanerophytes)	52	36	27	128	184
H (hemicryptophytes)	55	26	8	307	515
G (geophytes)	15	7	6	120	218
Ch (chamaephytes)	16	9	3	71	114
T (therophytes)	34	20	1	323	512
I (hydrophytes)	0	0	0	2	5
Habitat					
Temperate woodland	25	7	9	x	x
Maquis and garrigues	34	17	12	x	x
Mediterranean woodland	24	9	10	x	x
Arid environments	24	19	9	x	x
Along rivers and humid sites	20	3	4	x	x
Cultivated land	36	24	7	x	x
Uncultivated land	69	32	9	x	x
Maritime environments (dunes, alophilous rocks)	5	6	2	x	x
Walls, rocks	25	21	5	x	x

### Preliminary analysis of data

Plants listed in the three datasets (DTS1, DTS2, DT3) were checked for their presence in the two floras (recent and historical) of the area (Table 3). This operation was important for comparing species/families in each dataset that were actually listed in the floras for the following statistical analyses [e.g., 21 species listed in DTS1 are not mentioned in the historical flora (FL2)].

**Table 3:** Number of species and families in the three datasets (DTS1, DTS2, DTS3) in relation to the different floras (FL1 and FL2)

Categories	DTS1	DTS2	DTS3
Total taxa	176	102	45
Without subspecies	168	96	45
N° of families selected in FL1	63	44	28
N° of species selected in FL1	168	96	45
N° of families selected in FL2	61	52	27
N° of species selected in FL2	147	88	42

**Fig. 3:**
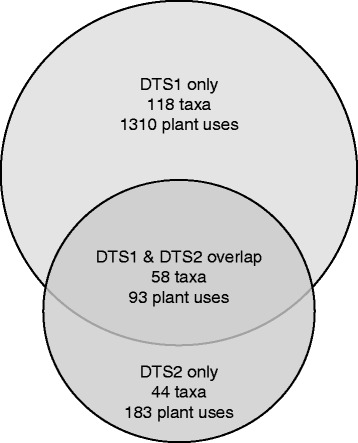
Medicinal plants (DTS1 and DTS2). Number of shared plant uses and taxa. Circles are proportional to the number of taxa

The medicinal floras share some similarities, but also several differences (both for species and plant uses). The medicinal flora of DTS1 includes 176 taxa (Table 3) belonging to 63 families, while the medicinal flora of DTS2 includes 102 taxa (2 taxa are at genus level) belonging to 44 families. These two datasets are only partially overlapping (Fig. 3).

The chorology, the life form and the habitat (according to Pignatti [66]) of all species (in DTS1, DTS2, DTS3) were recorded. The habitat of each species was reported in order to identify the habitats where it is possible to find more useful species as the same species may grow in more than one habitat. Places of gathering mentioned by informants were not considered since they were too generic, or not available.

The uses of plants for handicraft production, domestic and agro-pastoral practices in the Amalfi Coast were compared to other similar plant uses in Italian regions in order to seek points of convergence or divergence.

### Statistical analysis

In order to test whether a specific family is over or under-used in the Amalfi Coast we applied three different statistical methods to the three datasets (DTS1, DTS2, DTS3). In doing these analyses, we had to consider that:

1. The number of taxa in each family is different whether we count the number of taxa by considering or ignoring subspecies (difference is especially remarkable for floras, FL1, FL2).
2. The relative number of taxa in each family is different depending on how many taxa are listed in a given flora (Table 3).

Under these two different conditions, it was possible to generate floras and datasets with different numbers of elements, and different combinations of datasets within the floras. We compared the differences between the different datasets and floras but also compared results between the different statistical methods. We analyzed the studentized residuals from a linear regression [33], we compared proportions via binomial tests [2] and we developed confidence intervals around proportions using the Bayesian approach with a standard Uniform prior distribution [34]. We used the following data:

- DTS1 – DTS2 – DTS3: total number of taxa in each family considering FL1 (subspecies were not included).
- DTS1 – DTS2 – DTS3: total number of taxa in each family that were effectively listed in FL2 (subspecies were included).

Using linear regression, the total number of selected species *per* family was regressed on the total number of species in the same family in the area. The hypothesis underlying this method is that the “regression analysis gave a number of species that might be utilized as medicines in each family if they were selected at random” [72]. The over or under-used families were identified when the studentized regression residuals of the fitted line laying in the extreme tails of the Student’s t-distribution. While in Moerman et al. [15] the raw residuals are used, we computed here studentized residuals in order to compare them with the appropriate quantiles of the Student’s t distribution. Species with studentized residual falling into the lower (or upper) 5 % tails of the appropriate Student’s t distribution were considered as under-used (or over-used).

The binomial test is based on the assumption that medicinal species are allocated within a family according to the proportion of medicinal species in the flora [2]. We tested if the proportion selected within a family was proportionally more or less compared to the average proportion of the dataset. When the within-family proportion of selected species was statistically less (more) than the overall average proportion of the dataset, then the family was considered under-used (over-used).

The Bayesian approach is based on the assumption that the overall average proportion of selected plants in the flora is not observed without error; this being especially relevant for small datasets [34]. Using an uninformative Uniform (0,1) prior distribution, we compared the 95 % posterior credible interval of the average proportion selected in the overall dataset with the corresponding credible intervals for each family. If the credible interval for a family fell outside and left (right) of the credible interval for the overall dataset, the given family was considered under-used (over-used).

We then compared the percentage agreement among these three analyses. This allowed us to investigate the agreement among the results under different methods (linear regression, binomial or Bayesian) and under two different flora. The application of these methods is based on the assumption that the main factor that drives the selection of medicinal plant species is phylogeny (plants in the same family have closer relationships and will all be preferentially selected/avoided).

We investigated the effect of sample size on the ability to conduct a meaningful hypothesis test for over- and under-use. Our simulations were based on the datasets and floras identified in this paper, and showed whether a researcher would have the power to reject a null hypothesis for the observed sample sizes typical of ethnobotanical research studies. We identified the minimum number of taxa within a family that would be required to identify over-use and under-use given the three population proportions observed for FL1 and FL2.

We ran chi-square statistical analyses to test whether different selection criteria other than taxonomy may exist. Because a subset of cell counts were <5, we adjusted our *p*-values using 2000 replicate Monte Carlo simulations. In this analysis, we considered the taxonomic group according to APG III, the chorological form, the life form, and the habitat of each species (according to Pignatti [66]) in datasets DTS1, DTS2, DTS3 (see Table 2). Data were organized in contingency tables where the counts of the use versus the explanatory variables (taxonomic group, chorology, life form and habitat) were displayed. As contingency tables had dimensions greater than 2 × 2, we investigated the Pearson’s residuals for empirical evidence of departures from the null hypothesis of independence. All analyses were implemented using the software R [73].

## Results

There were 45 plant species used for handicraft production, domestic and agro-pastoral practices on the Amalfi Coast, with 95 different plant uses. These plant uses are detailed in the Additional file 1 (selected plant part, specific present or obsolete plant use, and the number of citations are also provided). Moreover, in the Additional file 1 we also report the similarity of each specific plant use with uses in other Italian regions. Among these plant uses many (62 %) are shared with other Italian regions while a rather high percentage of plant uses (38 %) seem to be typical of the Amalfi Coast. For example, *Spartium junceum* L. is used to make brooms in Calabria, in other areas of Campania, Lucania, Marche, Sicily, Trentino and Tuscany, while *Fraxinus ornus* L. subsp. *ornus* is used to make handles of farmyard utensils (Abruzzi, other areas of Campania, Marche, Molise, Sardinia and Sicily). Some plant uses seem to be typical of the Amalfi Coast, such as the use of *Thymelaea tartonraira* (L.) All. to make brooms to brush courtyards or the use of *Polystichum setiferum* (Forssk.) Moore ex Woyn for covering lemon orchards.

Among the species used for handicraft production, domestic and agro-pastoral practices (DTS3), many species are wild (30), some are cultivated in orchards (8) and seven are cultivated but can grow wild (probably to guarantee their availability). Wild plants are gathered in different kinds of habitats (Table 2). Medicinal plants (DTS1, DTS2) are generally gathered in uncultivated land. By analyzing the life form spectrum of ethnobotanical plants, Phanerophytes are predominant both in non-medicinal (60 %) and medicinal uses (30 %). Hemicryptophytes are more frequently used for non-medicinal uses, while Chamaephytes are preferred for medicinal uses. Considering the chorotype of ethnobotanical plants (DTS1, DTS2, DTS3), it is possible to highlight a prevalence of Mediterranean plants (35 % excluding repetitions across datasets). Many plants have a wide distribution (15 %) or a Eurasian areal (14 %). Considering the historical flora (FL2), Mediterranean plants constitute the 42 % and the Eurasian species the 19 %; thus, percentages of the ethnobotanical flora partially reflect the chorological spectrum of the whole flora (FL2).

### Statistical analysis

**Fig. 4:**
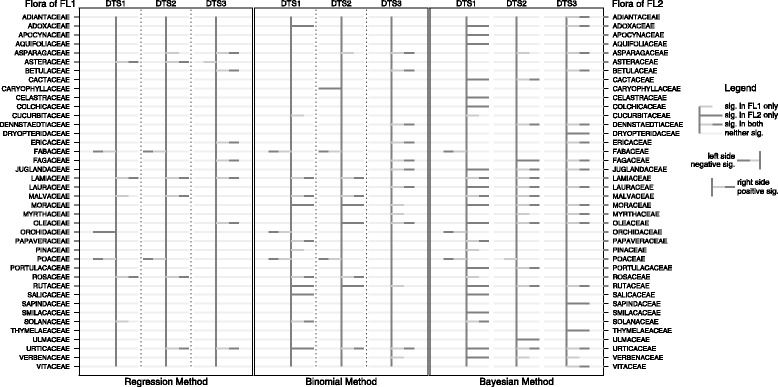
Linear regression, binomial method and Bayesian approach applied to the three datasets (DTS1-3) in relation to FL1 and FL2. Only families with over or under-use are showed. Results show significance in over or under-uses under different statistical tests. A darker line to the right indicates significant over-use of the plant family, while a darker line to the left indicates significant under-use. When both flora indicate similar results, the darker lines of each flora overlap with the line representing FL2 longer than that of FL1

The comparison of the three statistical methods to the three datasets (DTS1, DTS2, DTS3), in relation to the two floras FL1 and FL2, is reported in Fig. 4 (see also Additional file 2). Using FL1 and the medicinal dataset DTS1, the over-used families in the three statistical methods are Lamiaceae, Malvaceae, Rosaceae, Solanaceae, while the under-used families are Fabaceae, and Poaceae. Using FL2 and DTS1, we found over-use only in just one family, Lamiaceae, and found under-use in three (Fabaceae and Poaceae as in FL1, but also found Orchidaceae was under-used). Using FL1 and medicinal dataset DTS2, the over-used families according to all three statistical methods are Asparagaceae, Lamiaceae, Malvaceae and Urticaceae, with just one under-used family (Poaceae). Using FL2 and DTS2, we found over-use in three of the same families (Lamiaceae, Malvaceae and Urticaceae), and no under-used families. In both FL1 and FL2, and dataset DTS3 we found 6 over-used families similarly across all three statistical methods (Asparagaceae, Betulaceae, Ericaceae, Fagaceae, Oleaceae and Urticaceae) and no under-used families. In general, the floras FL1 and FL2 shared some agreement between medicinal dataset families identified as neither under- nor over-used, and similarities between under- and over-used families (e.g., Lamiaceae was identified as over-used in FL1 and FL2, DTS1 and DTS2). Overall, we found more families identified using DTS1 in FL1 (subspecies not included) compared to FL2 (subspecies included), and more non-medicinal dataset (DTS3) families identified in FL2 compared to FL1.

**Fig. 5:**
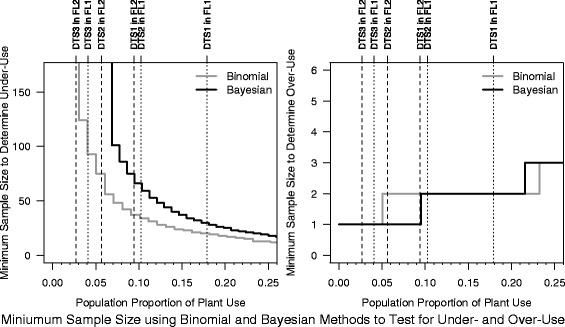
Results of a power analysis that compares the minimum sample size of taxa within a family that would be required to reject the null hypothesis in favor of concluding significant under- or over-use using the binomial and Bayesian methods. The vertical dashed lines depict the average proportion for DTS1, DTS2, DTS3 for floras FL1 and FL2. The step function is the minimum sample size required to statistically define when a plant family is under- or over-used. The location where the vertical line intercepts with the step function identifies the minimum within-family number of taxa required to statistically confirm under- and over-use for that dataset and flora. The left panel shows there is no power using the binomial or the Bayesian method to detect over-use in DTS3 and FL1 (i.e., we can never conclude that families in these datasets are statistically under-used). On the other hand, the right panel shows that very few within family taxa are required to have the power to statistically conclude over-use (all datasets, all flora discussed here require two or less taxa per family), and the Bayesian method is particularly sensitive in the region of datasets DTS1, DTS2 in FL2

**Fig. 6:**
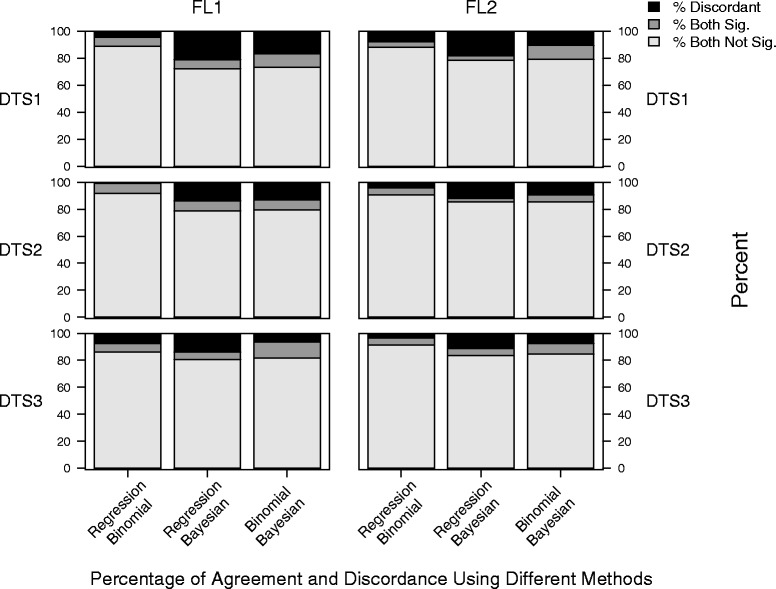
Percentages of agreement and discordance among the results obtained using different methods (linear regression, binomial method and Bayesian approach), with fixed floras. Agreement is when the significance is the same (both not significant, both significant and in the same direction)

In Fig. 5, we show the results of a power analysis that compares the minimum sample size of taxa within a family that would be required to reject the null hypothesis in favor of concluding significant under- or over-use using the binomial and Bayesian methods. The power analysis for the residual analysis from a linear regression model was not included as it is not a test of proportional selection. The power analysis results in Fig. 5 show there is no power using the Bayesian method to detect under-use in DTS2, DTS3 for FL2, or DTS3 for FL1 (and we must conclude no families in these datasets are statistically under-used). In general, the left panel shows that large sample sizes are required before the test is powered to statistically determine under-use, and shows the binomial approach is more powerful in determining under-use than the Bayesian method. For example, the right-hand-most vertical line shows the minimum sample size is 20 and 30 species within a family for the binomial and Bayesian methods to have sufficient number of species in them to have the statistical power to determine under-use. If we refer to the bottom panel of Fig. 2 we see that 16 and 11 families (in the top panel) would have the power to determine under-use using the binomial and Bayesian methods. These tests require much smaller sample sizes to be powered to determine over-use (right-hand panel; Fig. 5), and this is the reason behind our statistical results finding more families that are over-used than under-used. This is accentuated for the Bayesian method between population proportions of .05, and .093 which requires only one species in the family for a valid test, while the binomial method requires two. This is important in our study as the Bayesian analysis is able to reject the null hypothesis if that one species within the family is “used” and conclude over-use, whereas the binomial requires two. For example, we found that no families could be identified as over-used using binomial tests for DTS1 or DTS2, FL1 or FL2, whereas there are many identified using the Bayesian method. For this reason, there are far more determinations of over-use using the Bayesian method compared to the binomial method (Fig. 6). It is worth noting that no method has enough power to identify under-use when the overall use is already small (e.g., DTS3).

Figure 6 compares results between the two floras using the three statistical methods, and shows that there are differing conclusions from FL1 compared to FL2 (% discordant). In addition, this figure shows that the Bayesian method is most discordant from the residual regression analysis. This suggests again that the differences in datasets as well as choice of statistical method would affect the results and interpretation of the families within a dataset and flora.

**Fig. 7:**
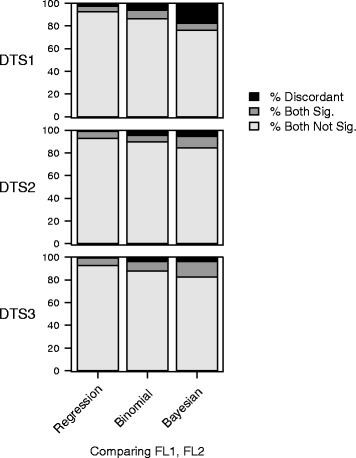
Percentages of agreement and discordance using floras FL1 and FL2 among the results obtained using the three statistical methods

**Fig. 8:**
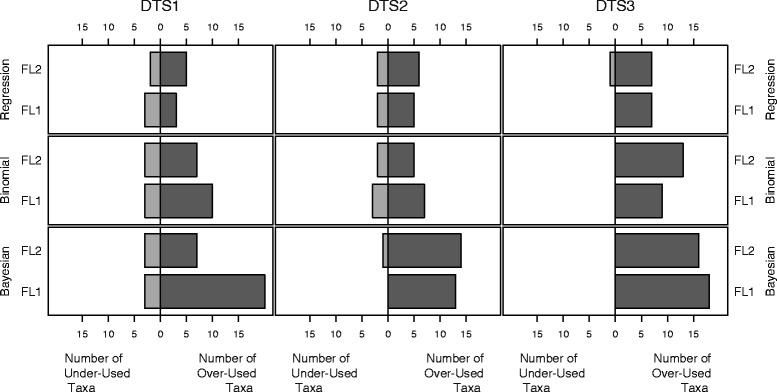
Number of under-used or over-used families using the different methods (linear regression, binomial method and Bayesian approach), floras (FL1 and FL2) and datasets (DTS1, DTS2, DTS3)

In Figs. 7 and 8, we show that for the datasets and floras of the Amalfi Coast, the Bayesian method identifies the highest number of over-used and under-used families, while linear regression identifies the lowest number (the “least common set of families” across the three methods).

The percentages of agreement (Figs. 6 and 7) among the different datasets and cases showed that:

- Results of statistical analyses are variable in relation to the combination of datasets, leading to different possible interpretations of data.
- In general the percentage of agreement is higher for FL1.
- As it is possible to observe in Fig. 6, the best agreement is reached by the regression and binomial methods for DTS2 with FL1.
- The lowest percentage of agreement in relation to methods is between the regression and Bayesian approach, for DTS 1 in FL2.

**Fig. 9:**
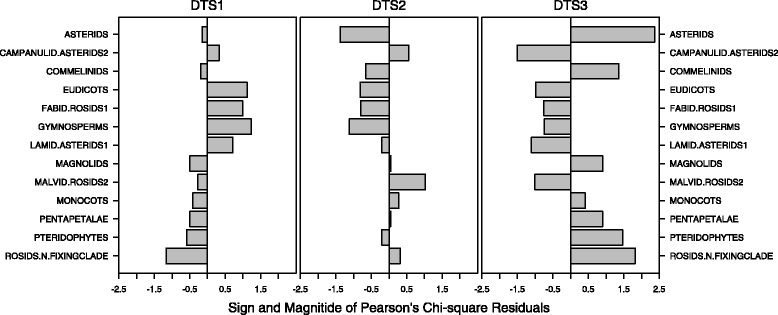
Plots of Pearson’s residuals for phylogeny, life form and habitat for DTS1, DTS2 and DTS3

The chi-squared test of independence yields significance for phylogeny, life form and habitat but not for chorology (*p*-value < 0.05). The Pearson’s residuals for phylogeny, life form and habitat are provided in Fig. 9, where the magnitude of the residual indicates a departure from the expected count. For DTS3, these analyses showed a non-random selection for plants in the Asterids taxon, woody (Phanerophytes) or growing in Temperate woodlands. Medicinal plants are more commonly selected within Hemicryptophytes (DTS1) and Therophytes (DTS2), growing in riverine (DTS1) and rocky (DTS2) habitats.

## Discussion

The number of plant uses in the Amalfi Coast is still high (in comparison to other Italian areas [74]) even if many are disappearing, and the cultural erosion is especially evident for younger generations [62]. It seems also that medicinal uses of plants are changing: there are some differences between DTS1 and DTS2 but these differences could be due also to the slightly different area of study and/or to the methods of data gathering.

Uses for handicraft production, domestic and agro-pastoral practices are various and some are reported also in other Italian areas. However, the lack of bibliography for several regions did not allowed us to define a clear pattern of diffusion of these uses, while this same analysis was easier for medicinal plants (see Savo et al. [62]). However, it was possible to highlight that in regions where a plant grows and where there are more studies, it is very likely that this plant would be used for handicraft production, domestic and agro-pastoral practices. This could mean that, at least for this category of use, plants might be chosen among the ones available, and then eventually for their intrinsic features (woody, flexible branches, smell, etc.). These features or “technical” qualities seem to be important on the Amalfi Coast, since many plants are gathered in the woodland, which is not always close to houses or fields.

### Patterns in plant use depends on different factors and on the kind of available data

The use of a plant depends on different factors. In many cases, plants which are abundant are more likely to be used [75] but plant uses might also be affected by the history and culture of the local population and by the degree of isolation from, or connection with, other cultures [76]. In Italy, some plants are widespread and are used in similar ways in many regions [74]. However, the different ways in which people use plants are complex and dynamic and the understanding of these processes is still rudimentary [77]. Potentially, both ecological and cultural factors may affect the traditional use of a plant in a dynamic and unique process.

Several hypotheses and theories have been expressed to explain patterns in plant selection. These have been explored and tested using different methods of analysis over the last years. However, before it is possible to perform these analyses, it is important to make some considerations on the data on which perform the tests. We found out that differences in floras affected our inference about preference and avoidance in use of plant families. In this paper, we observed several additional (non-statistical) problems in analyzing data. In order to define if plants are preferentially selected or avoided it is necessary to have a complete flora of the area or to analyze ethnobotanical lists of plants excluding species that are not listed in the flora. However, in this last case, culturally important plants may not be considered in the analysis: an example is given by cultivated plants (which are less in FL1 than FL2), that are not gathered in the wild, or by plants used by migrants, that are imported as dried preparations. Considering a recent flora or a complete flora (including historical data) for the analysis of data can change the results, since the two floras differ by more than 500 taxa (some plants have gone extinct, some plants have been introduced, etc.). Even the inclusion or the exclusion of subspecies led to differences of 85 taxa and 17 taxa in the historical (FL2) and in the recent flora (FL1) respectively. Moreover, the updating of the recent flora (FL1) to the very recent nomenclature has led to the elimination of 11 taxa (the flora originally had 966 taxa). This suggests that if the results and performances of the different methods vary depending on the kind of data available, the best method is dependent on the best available data that matches the study objectives.

The number of plant families in each dataset is different as well as the number of plant families in the floras (FL1 and FL2). These differences are due to different background factors, which are generally independent from the choices of researchers. However, these differences lead to different results (and interpretations) when looking for over-used or under-used families in a certain flora, and in many cases it can be particularly difficult due to lack of statistical power to determine under-use when there are limited number of taxa within a plant family.

In some cases, plants have uses tied to traditions, religion and ancient cultural practices. For example, the use of *Prunus avium* for pieces of furniture for brides, or the use of *Myrtus communis* branches to decorate wreaths of flowers. In Morocco these wreaths of flowers and *Myrtus* are placed on graves, while they are generically used in churches in Greece [78]. This plant seems to be related to religious uses in many countries even if it is not always specified how. Some plant uses are very ancient, since their scientific name is related to their use. For example the use of *Spartium junceum* (the name *Spartium* comes from the Greek Σπαρτον = rope) for its fibers: Phoenicians, Romans, and Greeks in fact used them to make sails [79]. Also the word *Ampelodesmos* originates from two old Greek cognales *àmpelos* (vine) and *desmòs* (tie) [80] and in Southern Italy it is widely used for tying plants to stakes [74].

### Statistical analysis

Our first objective was to test to whether differences in the selected datasets and analytic methods affected the interpretation of results. In a statistical sense, the “best” analysis is the one that either makes or violates the fewest statistical assumptions, while best from an anthropological perspective is the one that gives more consistent results. The analysis of three datasets (DTS1, DTS2, DTS3) and two floras using the three different statistical methods (linear regression residual analysis, binomial and Bayesian) gave different results for under-used or over-used families (Fig. 8). If the input data to the tests are the same, then these differences can be attributed to differences in tests and/or differences in the power of the tests. Without forcing the intercept of the linear regression through zero, the standardized residuals of the linear regression is testing a different hypothesis than the binomial and Bayesian approaches. Differences in determining under- and over-use in the same datasets and flora between the binomial and Bayesian approaches were merely a result of statistical power.

Differences between datasets and floras affected results for the Asteraceae family (and other families) but not for the families of Lamiaceae and mostly Malvaceae (with a single exception). This is rather interesting since the extensive use of Asteraceae and Lamiaceae as medicinal plants is reported in several ethnobotanical studies (e.g., [81, 82]). Asteraceae species are also commonly consumed as food plants [83] and this could affect their selection and potential use as medicinal plants. Differences may be generated by an evolution of plant uses, the period and method of gathering of data and/or to the flora of reference. The use of different floras (including subspecies or not) is not irrelevant for an ethnobotanical discussion, in the light of ethno-classification of species (e.g., in the case of over-differentiation [84]). Datasets were also analyzed including or excluding species that are not listed in the floras (FL1 or FL2): species could have been not well determined or taxonomy could have changed the classification (especially important if analyzing old floras, as for example in Dal Cero et al. [32]). Species that are not listed in the flora could also have been purchased or are only cultivated. The number of these species may be relevant when analyzing, for example, ethno-floras of migrant communities [85]. In general, the regression method is the least sensitive method, but over these different conditions (changing flora datasets) gave less variable results. On the other hand, binomial or Bayesian methods gave more variable results, but they could be used depending on your interest in over *vs* under-use as the Bayesian method is more sensitive to over-use, and the binomial method is more sensitive to under-use.

Our second aim was to test if the selection of plants for medicine or handicraft production, domestic and agro-pastoral practices is based on phylogeny, or other criteria (chorology, life form and habitat). In our case study, plants seem to be selected for more than one reason. There is a preference for woody plants (Phanerophytes) for handicraft production, domestic and agro-pastoral practices, which could be expected (many plants are used to build tools and instruments). Regarding medicinal plants, there is also a slight preference for herbaceous plants (Hemicryptophytes and Therophytes). This could be explained by the fact that many medicinal plants are weeds. Herbs and weeds tend to have a high content in secondary compounds since they are evident and appetizing for plant eating animals [86]. De Almeida et al. [87] hypothesize that annual plants should contain more secondary compounds than perennial plants, so the majority of medicinal plants should be annual, but in their study as well as in ours, this combination (annual-medicinal) is not strikingly significant. Herbaceous plants and weeds could also be selected for their availability and proximity, indeed, the riverine and rocky habitats in the area are in close proximity to the hamlets.

## Conclusions

The understanding of the reasons that drive people to select plants in a certain area is still rudimentary. Our study shows that selection criteria for plants (including medicinal plants) could not be limited to phylogeny, and it is likely that plants are selected for multiple different reasons. Finally, the comparison of different statistical methods (regression, binomial and Bayesian) under different conditions (different floras) led to the conclusion that the selection of the method is dependent on the best available data and the aims of the study. There are several differences in over-used families using the different methods, and this could lead to conflicting conclusions about plant selection criteria in a given area.

Developing insights about the complexity of cultural evolution and factors that drive changes is central to understanding the human experience and roles of environmental opportunities and constraints that are presented by plants. This work contributes to emerging theory about human interactions with plants specifically by helping researchers to focus their efforts and thoughtfully match analytical methods with results. We hope to see publications that show that researchers have considered the methods of analysis before collecting their data rather than fishing for analytical methods after collecting data.

## Additional files

Additional file 1:
**Species (and their plant parts), with vernacular names, currently or previously used for handicraft production, domestic and agro-pastoral practices (with details on their specific uses), the related number of citations and the eventual similarities with uses recorded in other Italian regions.** Species are listed in alphabetical order (of the family). Additional file 2:
**Linear regression, Binomial method and Bayesian approach applied to the three datasets (DTS1-3) in relation to FL1 and FL2 showing all the families.** A darker line the right indicates significant over-use of the plant family, while a darker line to the left indicates significant under-use. When both flora indicate similar results, the darker lines of each flora overlap with the line representing FL2 longer than that of FL1.
