# Supplementary material for: Plant selection for ethnobotanical uses on the Amalfi Coast (Southern Italy)
Source: J Ethnobiol Ethnomed. 2015 Jul 15;11:58. doi: 10.1186/s13002-015-0038-y (PMC4508904; doi:10.1186/s13002-015-0038-y)
Supplement: Additional file 1: — Species (and their plant parts), with vernacular names, currently or previously used for handicraft production, domestic and agro-pastoral practices (with details on their specific uses), the related number of citations and the eventual similarities with uses recorded in other Italian regions. Species are listed in alphabetical order (of the family). [file 13002_2015_38_MOESM1_ESM.docx]

Additional file 1

| **Species (vernacular name) and Family** | **Plant part used** | **Use category and sub-category** | **Use description** | **N° of citations** | **Currency of uses** | **Similar uses in other Italian regions** |
| --- | --- | --- | --- | --- | --- | --- |
| **Adiantaceae** | | | | | | |
| *Adiantum capillus-veneris* L. (“*capelli e jenere*”) | Fronds | DOM-Ornamental | Fronds are added to flower bouquets or are used to decorate the crèche | 5 | ** |  |
| **Adoxaceae** | | | | | | |
| *Sambucus nigra* L. (“*saucco*”) | Wood | HAND | The wood is carved into canes | 1 | * | Sardinia [A1] |
|  | Juice of the fruit | DOM-Dye | The black juice of fruit was used to prepare a kind of ink | 1 | *** | Abruzzi [A2,A3,A4]; Campania – Cilento National Park [A5,A6]; Latium [A7, A8]; Marche [A9,A10]; Sardinia [A1] |
|  | Fruit | AGR-Other | Branches with fruit are used to attract birds | 1 | *** |  |
| **Apiaceae** | | | | | | |
| *Foeniculum vulgare* Miller (“*finocchietto*”, “*finocchio selvatico*”, “*finucchiu*”) | Plant, Fruit | AGR-Scent | The plant or fruit are used to prepare a decoction, with other plants, which is then used to wash, clean and scent barrels before using them. The process is called “*cavara*” | 4 | ** | Liguria [A11]; Marche [A9,A10,A12]; Sardinia [A1]. This use is mentioned also in Amalfi [A13] in which are reported rather ancient traditional uses of the Sorrento Peninsula |
|  | Plant | AGR -Other | Wet plants were put under tomatoes in fruit boxes to increase their weight | 1 | *** |  |
| **Asparagaceae** | | | | | | |
| *Agave americana* L. (“*sambruino*”, “*agavi*”) | Fibers | DOM-Fibers | Fibers were used to obtain yarn which was then used to sew sacks | 3 | *** | Apulia [A14]; Campania – Ischia [A15]; Latium [A16,A17]; Lucania [A18,A19]; Sicily [A20,A21] |
| *Asparagus acutifolius* L. (“*asparagi*”) | Branches | DOM-Ornamental | Thorny branches are used to decorate the crèche | 1 | * | Sicily [A21,A22,A23] |
| *Ruscus aculeatus* L. (“*scannasorici*”) | Plant | DOM-Repellent | This plant is put on beams where salami and cheese are hanged | 3 | ** | Calabria [A24]; Campania – Cilento National Park [A6]; Latium [A7,A8]; Lucania [A18]; Molise [A25]; Sardinia [A1]; Trentino [A26]; Tuscany [A27] |
|  | Plant | DOM-Handicrafts | The plant was used to make brooms | 1 | *** | Abruzzi [A2]; Latium [A7,A8,A28]; Lucania [A18,A29]; Sardinia [A1,A29]; Sicily [A21,A23] |
| **Aspleniaceae** | | | | | | |
| *Ceterach officinarum* Willd. (*“pandosco”*) | Fronds | DOM-Ornamental | Fronds are used to decorate the crèche | 3 | * |  |
| **Asteraceae** | | | | | | |
| *Cynara* *cardunculus* L. subsp. *flavescens* Wiklund  (“*carciofo paesano*”) | Plant | AGR-Horticulture | A clay blow is put on artichoke flowers to keep the bracts closed | 1 | * |  |
| **Betulaceae** | | | | | | |
| *Alnus cordata* (Loisel.) Duby ("*autano*") | Wood | DOM-Firewood | The wood is used as firewood | 4 | ** | Lucania [A18] |
|  | Wood | AGR-Handicrafts | The wood is processed (*“sfilato”*) and used to make fruit boxes | 2 | * | Lucania [A18] |
|  | Wood | AGR-Wine- making | The wood was used to make wine barrels | 1 | *** |  |
| *Corylus avellana* L. (“*nocciole*”) | Nutshells | DOM-Firewood | Nutshells were used to stoke braziers and warming pans to make them last for a long time | 1 | *** |  |
| *Ostrya carpinifolia* Scop. (“*carpano*”) | Wood | DOM-Handicrafts | The wood is carved into kitchen utensils | 2 | * |  |
|  | Wood | DOM-Firewood | The wood is used as firewood | 1 | * | Campania - Cilento National Park [A6]; Lucania [A18] |
|  | Wood | AGR-Handicrafts | The wood is carved into farm tools | 1 | * | Abruzzi [A3]; Marche [A9]; Sicily [A21] |
| **Caryophyllaceae** | | | | | | |
| *Saponaria officinalis* L. (“*erba saponara*”) | Plant | DOM-Detergent | This plant was macerated in water for some days and then the water was used to wash clothes | 1 | *** | Calabria [A24]; Sardinia [A1] and in many other regions [A19] |
| **Dennstaedtiaceae** | | | | | | |
| *Pteridium aquilinum* (L.) Kuhn (“*spaccaprete*”) | Fronds | AGR-Other | Fronds are used to cover cherries when they are in baskets | 1 | * | Campania - Phlegraean Fields Regional Park [A30]; Latium [A7] |
|  | Fronds | AGR-Horticulture | Fronds were used to cover plants in the orchards | 1 | *** |  |
| **Dryopteridaceae** | | | | | | |
| *Polystichum setiferum* (Forsskal) T. Moore ex Woynar | Fronds | AGR-Horticulture | Fronds were used to cover lemon orchards | 1 | *** |  |
| **Ericaceae** | | | | | | |
| *Arbutus unedo* L. (“*sovero peloso*”, “*sorvo peloso*”) | Wood | HAND | The wood is carved into canes. The plant needs to be entire (with the roots). It is roasted in hot ashes until the trunk becomes easy to peel. This process is useful to retain the original color over time | 1 | * |  |
|  | Wood | AGR-Handicrafts | The wood was carved to obtain a part of the windlass that was used to carry trees from the mountain to the valley | 1 | *** |  |
| *Erica arborea* L. (“*olice*”, *“iolice*”, “*orgio*”) | Plant, Branches | DOM-Handicrafts | This plant is used to make brooms, especially the branches | 6 | ** | Campania - Cilento National Park [A5,A6] and Ischia [A15]; Sardinia [A1,A29]; Latium [A7,A8,A28,A29]; Lucania [A18,A29]; Sardinia [A1]; Sicily [A29,A31]; Tuscany [A27] |
|  | Branches | DOM-Firewood | Branches are used as a starter for fires | 1 | * | Campania - Cilento National Park [A6]; Sardinia [A1]; Sicily [A32,A33]; Tuscany [A27] |
|  | Roots | HAND | The wood of the root was used to make bowls or pipes | 2 | *** | Sardinia [A1]; Sicily [A31,A32,A34]; Sicily [A31] |
| **Fabaceae** | | | | | | |
| *Cytisus spinescens* C. Presl (“*spinzone*”) | Plant | AGR-Firewood | This plant was used as firewood in calcars | 1 | *** |  |
| *Spartium junceum* L. (“*ginestra*”) | Branches | AGR-Tying | Young branches are used to tied vegetables, especially tomatoes | 6 | ** | Calabria [A24]; Campania - Cilento National Park [A5,A6]; Latium [A28,A35]; Sardinia [A1]; Sicily [A21] and in other regions [A19] |
|  | Plant | DOM-Handicrafts | The plant was used to make brooms | 2 | *** | Calabria [A24]; Campania - Cilento National Park [A5,A6]; Lucania [A18,A36]; Marche [A37]; Sicily [A21]; Trentino [A26]; Tuscany [A38] |
| **Fagaceae** | | | | | | |
| *Castanea sativa* Miller (“*castagno*”) | Trunk | AGR-Viticulture | Trunks are used as stakes in vineyards and orchards. The tree should at least be 12-13 years old and cut down during winter | 25 | ** | Campania - Cilento National Park [A6]; Latium [A7,A28]; Lucania [A18]; Sardinia [A1]; Sicily [A21] |
|  | Wood, Branches | HAND | The wood is used to make pieces of furniture, stairs, doors, door and window frames. The tree should be at least 30-40 years old. Branches are used to make canes | 13 | ** | Abruzzi [A3]; Campania - Cilento National Park [A6]; Latium [A8,A35]; Lucania [A18]; Sardinia [A1]; Sicily [A21] |
|  | Branches, Root suckers | AGR-Tying | Branches, small trunks and root suckers are used as stakes for vegetables. Young root suckers (1 years old) were used to tie up faggots for calcars | 9 | ** | Sardinia [A1] |
|  | Wood | AGR-Wine- making | The wood is used to make barrels both for wine and food (as for example the anchovies in brine - the “*colatura di alici*”) | 7 | ** | Campania - Ischia [A15] and Phlegraean Fields Regional Park [A30]; Latium [A7,A8,A28,A35]; Lucania [A18]; Sardinia [A1] |
|  | Wood | DOM-Handicrafts | The wood is cooked and the fibers are stripped out (or the bark) to weave baskets. Few people still produce this traditional baskets. The town of Tramonti was famous for this product. Fibers are also woven to create chair seats | 7 | ** | Campania - Ischia [A15] and Phlegraean Fields Regional Park [A30]; Lucania [A18,A39]; Piemonte [A40]; Tuscany [A41] |
|  | Wood | DOM-Other | The wood is used for the wooden planking in buildings and to build wooden beam floors | 9 | ** | Abruzzi [A3]; Campania - Cilento National Park [A6] and Ischia [A15]; Lucania [A18]; Sardinia [A1]; Sicily [A21] |
|  | Wood | DOM-Firewood | The wood is used as firewood. In the past, it was possible to receive a piece of bread in exchange for a bundle of sticks | 4 | ** | Campania - Cilento National Park [A6] |
|  | Branches | AGR-Handicrafts | Branches are used to make handles of utensils. The wood of young trees was used to make fruit boxes | 6 | ** | Campania - Ischia [A15] |
|  | Wood | AGR-Fences | The wood is used to make fences | 3 | * | Sicily [A21] |
|  | Branches | AGR-Horticulture | Branches were used to cover orchards. They were gathered on the mountains in June and then carried down on shoulders | 1 | *** |  |
|  | Trunk, Wood | AGR-Other | Trunks were used to make lampposts. The wood was carved to obtain a part of the windlass that was used to carry trees from the mountain to the valley | 2 | *** |  |
| *Fagus sylvatica* L. (“*faggio”*) | Wood | DOM-Firewood | The wood is used as firewood | 1 | * | Latium ([A7]); Molise [A25] |
|  | Wood | HAND | The wood was used to make canes | 1 | *** |  |
| *Quercus ilex* L. (“*leccio*”, “*lecino*”) | Wood | DOM-Firewood | The wood is used as firewood and charcoal since it is resistant and lasts for long time in the fire | 13 | ** | Campania - Cilento National Park [A6]; Latium [A7,A8]; Lucania [A18]; Sardinia [A1]; Tuscany [A27] |
|  | Branches | AGR-Horticulture | Branches are used to cover lemon orchards and to protect lemons from “*libeccio”* (wind with salty air) | 6 | ** | Lucania [A18] |
|  | Plant | AGR-Ornamental | The plant is planted along the street for decoration | 1 | * |  |
| *Quercus pubescens* Willd. (“*quercia*”, “*cerza”*) | Wood | DOM-Firewood | The wood is used as firewood | 2 | * | Campania - Cilento National Park [A6]; Latium [A7,A8]; Lucania [A18]; Marche [A9] Molise [A25]; Sardinia [A1] |
|  | Wood | HAND | The wood is used to build tables | 1 | * | Molise [A25]; Sardinia [A1] |
|  | Wood | AGR-Wine- making | The wood was used to make wine barrels | 1 | *** | Latium [A7,A8]; Lucania [A18]; Marche [A9,A12] Sardinia [A1] |
| **Juglandaceae** | | | | | | |
| *Juglans regia* L. (“*noce*”) | Wood | HAND | The wood is used to make furniture, door and window frames | 4 | ** | Abruzzi [A42]; Campania - Cilento National Park [A6]; Latium [A8]; Lucania [A18]; Marche [A9,A12]; Molise [A25]; Piemonte [A40]; Sardinia [A1] |
|  | Wood, Nutshells | DOM-Firewood | The wood is used as firewood. Nutshells were used to stoke braziers and warming pans since they last for a long time | 2 | ** |  |
| **Lamiaceae** | | | | | | |
| *Clinopodium nepeta* (L.) Kuntze (“*nepeta*”) | Plant | DOM-Scent | Bouquets of the plant were used to scent linen | 1 | *** | Campania - Cilento National Park [A5]; Sardinia [A1] |
| *Lavandula angustifolia* Mill. subsp. a*ngustifolia* (“*lavanda*”, “*spigandosso*”) | Floral tops | DOM-Scent | Floral tops with the stems are tied together and used to scent linen | 6 | * | Abruzzi [A2]; Calabria [A24,A36]; Campania - Cilento National Park ([A5]) and Phlegraean Fields Regional Park [A30]; Latium [A4,A28,A43]; Liguria [A44]; Lucania [A18]; Marche [A9,A12,A45]; Sardinia [A1]; Trentino [A26] |
| *Micromeria graeca* Bentham subsp. *graeca* (“*spigandosso*”, “*erba di tosse*”) | Plant | AGR-Scent | The plant was added to boiling water that was put into barrels to wash and scent them. The process is called “*cavara*” | 1 | *** |  |
|  | Plant | DOM-Scent | The plant is used to scent linen | 1 | * |  |
| *Thymus longicaulis* C. Presl (“*timo”*) | Plant | AGR-Scent | This plant was boiled with fennel and the water was then used to clean and scent barrels | 1 | *** |  |
| **Lauraceae** | | | | | | |
| *Laurus nobilis* L. (“*alloro*”, “*lauro*”, “*o’lauro*”) | Leaves | DOM-Detergent | Leaves along with ash were put in hot water that was then used to wash clothes | 3 | *** | Latium ([A7]); Liguria [A44]; Sardinia [A1] |
|  | Leaves | AGR-Scent | Leaves are mixed to other plants to prepare a decoction that is used to wash, clean and scent barrels before using them. The process is called “*cavara*” | 2 | * | Abruzzi [A42]; Campania – Ischia [A15]; Sicily [A23] |
|  | Wood | HAND | The wood is used to make canes | 1 | * |  |
|  | Leaves | DOM-Other | Leaves are used to reduce the fat of large eels | 1 | * |  |
|  | Leaves | DOM-Scent | Leaves are used to scent soap and linen | 1 | *** | Piemonte [A40]; Sardinia [A1] |
| **Moraceae** | | | | | | |
| *Ficus carica* L. (“*fica*”, “*ficozze*”) | Latex | AGR-Cheese-making | The latex of young branches was used as rennet for cheese | 1 | *** | Abruzzi [A3,A42]; Campania - Cilento National Park [A5,A6] and Phlegraean Fields Regional Park [A30]; Latium [A7,A8]; Lucania [A18]; Sardinia [A1]; Sicily [A21,A23,A46,A47] and in other regions [A19] |
| **Myrtaceae** | | | | | | |
| *Myrtus communis* L. subsp. *communis* (“*mortella”, “mirtillo”*) | Branches | DOM-Ornamental | Leafy branches are used to decorate bouquets of flowers | 1 | * | Branches are used to decorate wreaths of flowers in Campania - Cilento National Park [A6]; Lucania [A18]; Sardinia [A1]; Sicily [A21] |
|  | Branches | DOM-Handicrafts | Flexible branches are used to weave baskets | 1 | * | Campania - Ischia [A15]; Latium[A16]; Lucania [A18]; Sardinia [A1] |
|  | Wood | HAND | The wood is carved into shepherd canes | 1 | * |  |
| **Oleaceae** | | | | | | |
| *Fraxinus ornus* L. (“*frasso*”) | Trunk, branches | AGR-Handicrafts | Small trunks and branches are used to make handles of farmyard utensils | 3 | * | Abruzzi [A2]; Campania - Cilento National Park [A6]; Lucania [A18]; Marche [A9,A12]; Molise [A25]; Sardinia [A1]; Sicily [A47] |
|  | Wood | DOM-Firewood | The wood is used as firewood | 3 | * | Latium ([A7]) |
|  | Trunk, Branches | AGR-Horticulture | Trunks may be used as stakes in orchards instead *of Castanea sativa* trunks. Branches are used as stakes for vegetables | 2 | * | Campania - Cilento National Park [A6]; Latium ([A7]); Lucania [A18]; Sardinia [A1] |
|  | Wood | HAND | The wood is used to make chairs and is carved into typical clogs | 1 | ** | Sardinia [A1] |
|  | Wood | AGR-Other | The wood was used to make jointer planes | 1 | *** |  |
| *Olea europaea* L. (“*olivo*”, “*aulivo*”) | Branches, Wood | DOM-Handicrafts | Branches and root suckers are used to weave baskets. The wood was carved into kitchen utensils | 3 | ** | Abruzzi [A42]; Calabria [A24]; Campania - Cilento National Park [A6] and Ischia [A15]; Latium [A16,A48]; Lucania [A18,A39]; Sardinia [A1]; Sicily [A21,A33,A34,A46,A49,A50,A51]; Tuscany [A27,A41] |
|  | Seeds | DOM-Firewood | Seeds of olives were used to stoke braziers and warming pans in order to make them last for a long time | 1 | *** |  |
| **Poaceae** | | | | | | |
| *Arundo mediterranea* Danin (“*libbano”*) | Leaves | DOM-Handicrafts | Leaves were used to make brooms. Leaves were weaved to make doormats but also the seats of chairs | 7 | *** | Campania - Cilento National Park [A6]; Latium [A7, A16,A17]; Lucania [A18,A29,A39]; Sardinia [A1,A29]; Sicily [A21,A22,A23,A29,A46] |
|  | Stem | DOM-Other | Stems were used to keep pizzas separated. Stems were used as rolling shutters | 2 | *** |  |
|  | Plant | DOM-Firewood | The plant was used to start fires | 1 | *** | Latium [A28,A35]; Lucania [A18,A39]; Sardinia [A1]; Sicily [A23] |
| *Cynodon dactylon* (L.) Pers. (“*gramegna*”) | Plant | DOM-Detergent | The plant was put in the same water that is used to wash clothes | 1 | *** |  |
| *Lagurus ovatus* L. | Plant | DOM-Ornamental | The plant is used to decorate dried flowers compositions | 1 | * | Sardinia [A1] |
| **Rosaceae** | | | | | | |
| *Cormus domestica*(L.) Spach (“*sorbo*”, “*sorvo*”) | Wood | DOM-Firewood | The wood is used as firewood | 1 | * |  |
|  | Wood | HAND | The wood was carved into a kind of clog | 1 | *** |  |
| *Prunus avium* L. (“*ceraso*”) | Wood | HAND | The wood is used to make pieces of furniture or carved into a kind of clog | 4 | ** | Abruzzi [A42]; Campania - Cilento National Park [A6]; Latium [A7,A8,A28]; Lucania [A18]; Marche [A9,A12]; Sardinia [A1]; Tuscany [A52]. In Abruzzi its wood is used to make chairs [A3] |
| **Rutaceae** | | | | | | |
| *Citrus limon* (L.) Burm. (“*limone*”) | Wood | DOM-Handicrafts | The wood was carved into handles of utensils | 1 | *** |  |
|  | Peels of the fruit | DOM-Scent | The peels of the fruit were used to scent the laundry water | 1 | *** | Sardinia [A1] |
| **Salicaceae** | | | | | | |
| *Salix alba* L. (“*salice*”) | Branches | AGR-Tying | Young branches are used to tie plants, especially grapes. Salix plants are often cultivated at the edge of vineyards | 20 | ** | Campania - Cilento National Park [A5,A6] and Phlegraean Fields Regional Park [A30]; Lucania [A18]; Sardinia [A1]; Tuscany [A41,A53] |
|  | Branches | DOM-Handicrafts | Branches are weaved to make baskets or chair seats | 5 | ** | Abruzzi [A4]; Campania - Cilento National Park [A5,A6] and Phlegraean Fields Regional Park [A30]; Latium [A7,A8;A35,A48]; Marche [A9,A12,A54]; Molise [A25]; Sardinia [A1]; Sicily [A21,A34,A50]; Tuscany [A41,A53;A55] and in many other areas [A19] |
|  | Wood | HAND | The wood was carved into typical clogs | 1 | *** |  |
| **Sapindaceae** | | | | | | |
| *Acer opalus* Mill. subsp. o*btusatum* (Waldst. & Kit. ex Willd.) Gams  (“*cucchiaro*”) | Wood | DOM-Handicrafts | The wood was carved into spoons and other kitchen utensils | 5 | *** |  |
|  | Wood | AGR-Pastoralism | The wood was carved into goat collars. They are closed when the wood is still wet, after that, it becomes rigid. It is necessary to protect the neck of animals | 2 | *** | Lucania [A18,A39,A56]; Sicily [A21] |
|  | Wood | DOM-Firewood | The wood is used as firewood | 1 | * | Lucania[A18,A39,A56] |
|  | Wood | HAND | The wood was carved into a sort of clogs | 1 | *** | Lucania [A18,A39,A56] |
| **Thymelaeaceae** | | | | | | |
| *Thymelaea tartonraira* (L.) All. (“*zampe di gallina*”) | Plant | AGR-Handicrafts | The plant was dried to make brooms to clean the courtyard | 1 | *** |  |
| **Urticaceae** | | | | | | |
| *Parietaria* sp. pl. *(Parietaria judaica* L., *Parietaria officinalis* L.) (“*parietaria*”, “*pardana*”, “*pardale*”) | Leaves | DOM-Detergent | Leaves are used to clean windows | 1 | * | Abruzzi [A4,A42]; Calabria [A24]; Campania - Phlegraean Fields Regional Park [A30]; Latium [A8]; Liguria [A11]; Marche [A9,A12,A54]; Sardinia [A1]; Sicily [A46]; Trentino [A26]; Tuscany [A55]; Umbria [A57] |
| **Verbenaceae** | | | | | | |
| *Aloysia citriodora*Palau (“*erba cedra*”, “*erba cedrina*”) | Plant | DOM-Scent | The plant was used along with *Laurus nobilis* to scent the laundry water. Bouquets of the plant are used to scent linen | 2 | ** | Latium [A7]; Sardinia [A1]; Sicily [A58] |
| **Vitaceae** | | | | | | |
| *Vitis vinifera* L. s.l. (“*vite*”, “*uva*”) | Plant | AGR-Viticulture | Local species of grapes (as the *Barbarella*) are used as rootstocks for grape cultivation | 1 | * |  |

**Abbreviations**: AGR= Agro-pastoral use; DOM: Domestic use; HAND=Handicrafts. *=Current use; **=Disappearing use; ***=Obsolete use.

**Notes: The sub-categoory classification follows Signorini et al. [A59]. In the use description, we used a past tense to indicate obsolete uses and the present tense for current uses.**

**References**

A1. Atzei AD: *Le piante nella tradizione popolare della Sardegna*. Sassari: C Delfino; 2003.

A2. Tammaro F: *Flora Officinale d'Abruzzo*. Chieti: Giunta Reg d’Abruzzo; 1984.

A3. De Simoni E, Guarrera PM: **Indagine Etnobotanica nella provincia di Teramo**. *Quaderni Botanica Ambientale Applicata* 1994, **15**: 3-10.

A4. Idolo M, Motti R, Mazzoleni S: **Ethnobotanical and phytomedicinal knowledge in a long-history protected area, the Abruzzo, Lazio and Molise National Park (Italian Apennines).** *Journal of Ethnopharmacology* 2010, **127**: 379–395.

A5. Scherrer AM, Motti R, Weckerle CS: **Traditional plant use in the areas of Monte Vesole and Ascea, Cilento National Park (Campania, Southern Italy)**. *Journal of Ethnopharmacology* 2005, **97**:129-143.

A6. Salerno G, Guarrera PM: **Ricerche etnobotaniche nel Parco Nazionale del Cilento e Vallo di Diano: il territorio di Castel San Lorenzo (Campania, Salerno).** *Informatore Botanico Italiano* 2008, **40:** 165-181.

A7. Guarrera PM: *Il Patrimonio Etnobotanico del Lazio Censimento del Patrimonio Vegetale del Lazio.* Rome: Quaderno n°1 Regione Lazio, Assess Cultura e Dipartim Biologia Veget, Univ “La Sapienza”, Tipar; 1994.

A8. Guarrera PM, Forti G, Marignoli S, Gelsomini G: *Piante e Tradizione popolare ad Acquapendente*. Acquapendente: Quaderni del Museo del Fiore n 2, Museo del Fiore; 2004.

A9. Guarrera PM: **Ricerche etnobotaniche nelle province di Macerata e di Ancona**. *Rivista Italiana EPPOS* 1981, **2:** 99-108.

A10. Taffetani F: *Rugni, Speragne e Crispigne Piante Spontanee negli usi del Territorio maceratese*. Macerata: Fondazione Cassa di Risparmio della Provincia di Macerata, Carima Arte srl; 2005.

A11. Maccioni S, Tomei PE, Rizzo A: **L'uso medicinale delle specie vegetali selvatiche e coltivate nella tradizione popolare della bassa Val di Magra.** *Memorie dell’Accademia Lunigianese di Scienze “Giovanni Capellini”* 1995, **LXIV-LXV**: 389-435.

A12. Guarrera PM: **Le piante nelle tradizioni popolari delle Marche.** *Erboristeria Domani* 2005, **293**: 52-59.

A13. Amalfi G: *Tradizioni ed Usi nella Penisola Sorrentina*. Palermo: Pedone Lauriel; 1890.

A14. Leporatti ML, Guarrera PM: **Contributo alla conoscenza degli usi tradizionali delle piante in Capitanata e Salento (Puglia)**. In: *Atti del 99° Congresso della Società Botanica Italiana*: *22-24 September 2004; Turin*. Edited by the Società Botanica Italiana; 2004: 285.

A15. Vallariello G: **Etnobotanica dell'Isola d'Ischia (Napoli, Italia).** *Delpinoa* 2003, **45:** 233-243.

A16. Novellino D: **An account of basket weaving and the use of fibre plants in the Mount Aurunci park (Central Italy).** In: *Proceedings of the Fourth International Congress of Ethnobotany (ICEB 2005). Ethnobotany: at the Junction of the Continents and the Disciplines*. Edited by Ertug, F (Ed): Ege Yayinlari, Istanbul; 2006: 317-326.

A17. Novellino D: ***Ampelodesmos mauritanicus* The role of *Ampelodesmos mauritanicus* and fiber plants in central Italy.** *Non-Wood news* 2007, **14**: 24-25.

A18. Salerno G, Guarrera PM, Caneva G: **Agricultural, domestic and handicraft folk uses of plants in the Tyrrhenian sector of Basilicata (Italy).** *Journal of Ethnobiology and Ethnomedicine* 2005, **1**: 2.

A19. Guarrera PM: *Usi e Tradizioni della Flora italiana. Medicina popolare ed etnobotanica*. Rome: Aracne Ed.; 2006.

A20. Raimondo FM, Schicchi R: *Il popolamento vegetale della Riserva naturale dello Zingaro*. Palermo: Aziende Foreste Demaniali; 1998.

A21. Arcidiacono S, Napoli M, Oddo G, Pavone P: **Piante selvatiche d'uso popolare nei territori di Alcara li Fusi e Militello Rosmarino (Messina, N-E Sicilia).** *Quaderni Botanica Ambientale Applicata* 2007, **18:** 105-146.

A22. Napoli M, Giglio T: **Usi popolari di piante spontanee nel territorio di Monterosso Almo (Ragusa).** *Bollettino dell’Accademia Gioenia di Scienze Naturali in Catania* 2002, **35**: 361-401.

A23. Arcidiacono S, Napoli M, Pavone P: **Piante selvatiche d'uso popolare nel territorio di Bronte (Catania).** *Quaderni Botanica Ambientale Applicata* 2003, **14:** 151-172.

A24. Passalacqua NG, De Fine G, Guarrera PM: **Contribution to the knowledge of the veterinary science and of the ethnobotany in Calabria region (Southern Italy).** *Journal of Ethnobiology and Ethnomedicine* 2006, **2:** 52.

A25. Guarrera PM, Lucchese F, Medori S: *L’uso Tradizionale delle Piante nell’Alto Molise*. Rome: Tipar Arti Grafiche: 2009.

A26. Pedrotti G, Bertoldi V: *Nomi dialettali delle piante indigene del Trentino e della Ladinia dolomitica Presi in esame dal punto di vista della botanica della linguistica e del folklore*. Trento: Monauni; 1930.

A27. Mearelli F, Tardelli C: **Maremma Mediterranea. Le piante e l’uomo**. *Erboristeria Domani*, 1995, **7/8:** 45–57.

A28. Guarrera PM: **Le piante nelle tradizioni popolari del Lazio**. *Erboristeria Domani* 2004, **281:** 53-62

A29. Nedelcheva AM, Dogan Y, Guarrera PM: **Plants traditionally used to make brooms in several European countries.** *Journal of Ethnobiology and Ethnomedicine* 2007, **3**: 20.

A30. Motti R, Antignani V, Idolo M: **Traditional Plant Use in the Phlegraean Fields Regional Park (Campania, Southern Italy*).*** *Human Ecology* 2009, **37:** 775–782.

A31. Raimondo FM, Lentini F: **Indagini etnobotaniche in Sicilia I Le piante della flora locale nella tradizione popolare delle Madonie (Palermo).** *Naturalista siciliano* 1990, **IV:** 77-99.

A32. Galt AH, Galt JW: **Peasant use of some wild plants on the Island of Pantelleria**. *Economic Botany* 1978, **32**: 202-226.

A33. Lentini F, Di Martino A, Amenta R: **La flora popolare dell’isola di Ustica (Palermo).** *Giornale Botanico Italiano* 1996, **129:** 167.

A34. Lentini F, Giani S, Amenta R: **L'uso popolare delle piante nelle isole Eolie (Sicilia).** *Acta Technologiae et Legis Medicamenti* 1995, **3**: 351-355.

A35. Guarrera PM, Lattanzi E: **Aspetti della Flora dei Monti Prenestini con osservazioni sulle piante officinali ed i loro usi popolari**. In *Aspetti naturalistici dei Monti Prenestini.* Edited by Angelici, FM. Gennazzano: Associazione Naturalistica "Orchidea" Regione Lazio, Assessorato Ambiente; 2001.

A36. Bernardo L: *Fiori e piante del Parco del Pollino, 2a edition*. Castrovillari: Prometeo; 2000.

A37. Guarrera PM: **Usi tradizionali delle piante in alcune aree marchigiane**. *Informatore Botanico Italiano* 1990, **22**: 155-167.

A38. Pieroni A: **Piante spontanee ed immaginario collettivo in Alta Garfagnana (Lucca): un centro di documentazione sulla cultura orale.** *Informatore Botanico Italiano* 1999, **31:** 183-189.

A39. Guarrera PM, Salerno G, Caneva G: **Indagini etnobotaniche nel versante tirrenico della Basilicata.** In: *Atti del 98° Congresso della Società Botanica Italiana: 24-26 September 2003; Catania*. Edited by Società Botanica Italiana; 2003: 138.

A40. Sella A: *Flora popolare biellese. Nomi dialettali, tradizioni e usi locali*. Torino: Edizioni Dell'Orso; 1992.

A 41. Beconcini P, Giusti ME, Venturelli G: *L'intrecciatura tradizionale in area* lucchese. Rome: MNATP, Quasar; 1984.

A42. Guarrera PM: **Usi tradizionali delle piante nel territorio della Majella**. In: *Fascicolo Monografico su Erbe e Piante Medicinali nella Storia e nelle Tradizioni Popolari Abruzzesi*. Edited by AAVV. Chieti; 1987: 17–44.

A43. Guarrera PM, Forti G, Marignoli S: **Ethnobotanical and ethnomedicinal uses of plants in the district of Acquapendente (Latium, Central Italy).** *Journal of Ethnopharmacology* 2005a, **96**: 429–444.

A44. Cornara L, La Rocca A, Marsili S, Mariotti MG: **Traditional uses of plants in the Eastern Riviera (Liguria, Italy).** *Journal of Ethnopharmacology* 2009, **125**: 16–30.

A45. Bellomaria B, Lattanzi E: **Le piante del territorio di Cupra Marittima (Marche) attualmente usate nella medicina popolare**. *Archivio Botanico e Biogeografico Italiano* 1982, **58**: 155-173.

A46. Lentini F, Raimondo FM: **Indagini etnobotaniche in Sicilia IV L'uso tradizionale delle piante nel territorio di Mistretta (Messina).** *Quaderni Botanica Ambientale Applicata* 1990, **1:** 103-117.

A47. Lentini F, Mazzola P: **Le piante utilizzate nella tradizione locale**. In *Il popolamento vegetale della riserva dello Zingaro*. Edited by Raimondo FM, Schicchi R. Palermo: Azienda Foreste Demaniali; 1998.

A48. Guarrera PM: **Fitoterapia, piante e credenze tradizionali relative alle piante nella provincia di Roma.** *Storia e Medicina Popolare* 1989, **7**: 3-38.

A49. Lentini F, Catanzaro F, Aleo M: **Indagini etnobotaniche in Sicilia III. L'uso tradizionale delle piante nel territorio di Mazara del Vallo (Trapani).** *Atti dell’Accademia di Scienze, Lettere e Arti di Palermo* 1988, 1-29.

A50. Lentini F, Di Martino A, Amenta R: **Contributo alla conoscenza della flora popolare dell’isola di Ustica.** *Quaderni Botanica Ambientale Applicata* 1994, **5**: 47-54.

A51. Lentini F: **The role of Ethnobotanics in scientific research. State of the ethnobotanical knowledge in Sicily**. *Fitoterapia* 2000, **71**: S83-S88.

A52. Tomei PE, Monti G, Onnis A: *Specie vegetali cultivate e spontanee di uso alimentare e medicinale nella tradizione popolare dell’alta Garfagna*. Pisa: Dipartimento di Scienze Botaniche dell’Università di Pisa, Pacini Ed; 1988.

A53. Tomei PE, Gaspari G: **Indagine sulle zone umide della Toscana XVI Le piante officinali dei bacini palustri della Toscana settentrionale**. *Atti della Società Toscana di Scienze Naturali, Memorie* 1981, **88**: 175-195.

A54. Bellomaria B: **Le piante di uso popolare nel territorio di Camerino (Marche).** *Archivio Botanico e Biogeografico Italiano* 1982, **58**: 1-27.

A55. Mambrini M, Vicarelli GB: *Piante officinali dell'Amiata Usi e tradizioni popolari*. Castell'Azzara: Cooperativa Agricola Forestale dei Comuni Amiatini; 1983.

A56. Guarrera PM, Salerno G, Caneva G: **Folk phytotherapeutical plants from Maratea area (Basilicata, Italy).** *Journal of Ethnopharmacology* 2005b, **99**: 367-378.

A57. Nardelli GM: *Cultura e tradizione. Demomedicina nell'alta Umbria*. Perugia: Provincia di Perugia; 1987.

A58. Catanzaro F: **Piante officinali dell'Isola di Pantelleria.** *Webbia* 1968, **23:** 135-148.

A.59. Signorini MA, Bruschi P, Camangi F, Guarrera PM, Pieroni A, Savo V. (2013). **Metodi della ricerca etnobotanica.** In *Etnobotanica, Conservazione di un patrimonio culturale immateriale come risorsa per uno sviluppo sostenibile* Edited by Caneva G, Pieroni A, Guarrera PM. Bari: EdiPuglia, Bari; 2013.
