# Supplementary figures and images for: Plant selection for ethnobotanical uses on the Amalfi Coast (Southern Italy)
Source: J Ethnobiol Ethnomed. 2015 Jul 15;11:58. doi: 10.1186/s13002-015-0038-y (PMC4508904; doi:10.1186/s13002-015-0038-y)

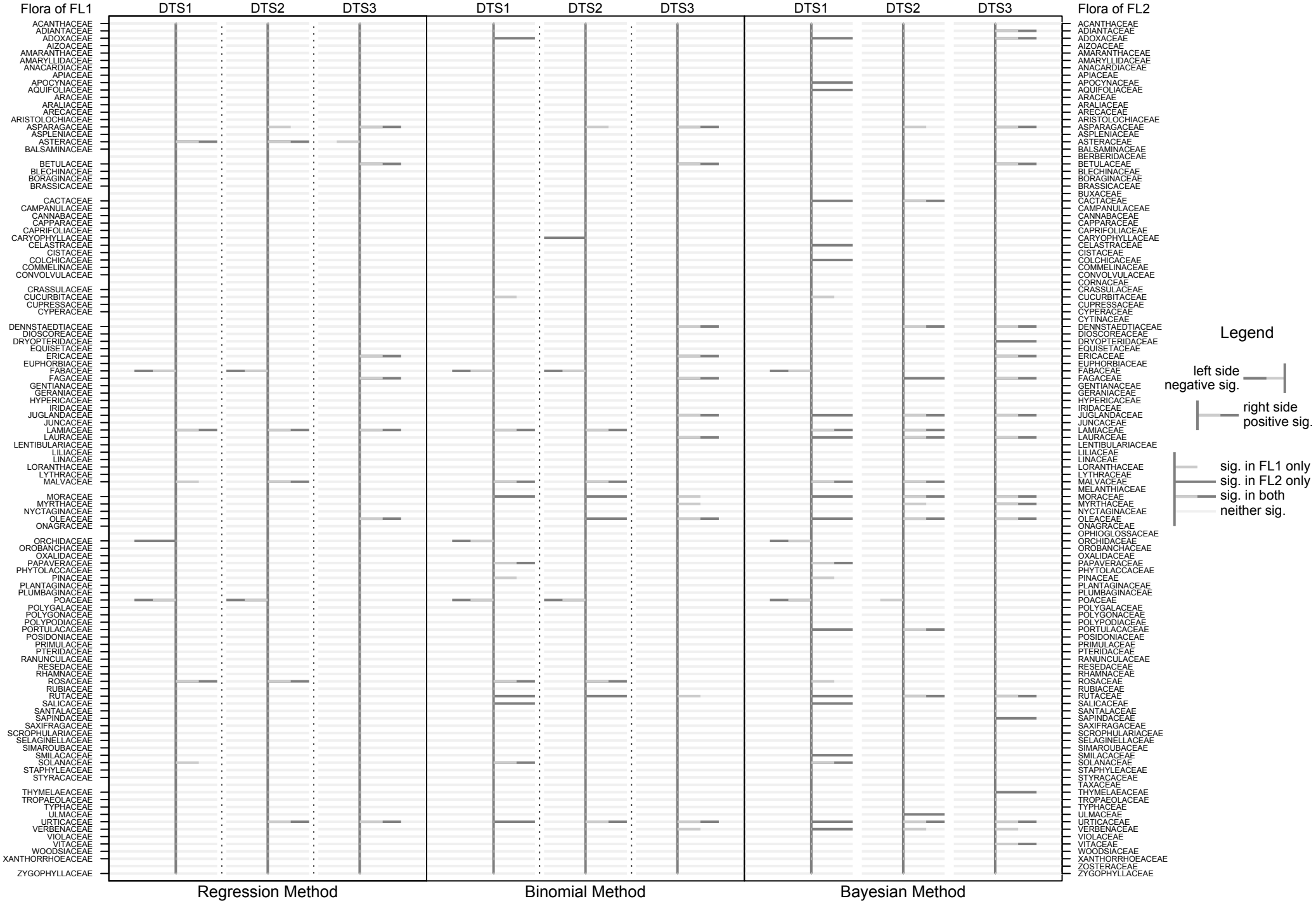

Supplement: Additional file 2: — Linear regression, Binomial method and Bayesian approach applied to the three datasets (DTS1-3) in relation to FL1 and FL2 showing all the families. A darker line the right indicates significant over-use of the plant family, while a darker line to the left indicates significant under-use. When both flora indicate similar results, the darker lines of each flora overlap with the line representing FL2 longer than that of FL1. [file 13002_2015_38_MOESM2_ESM.pdf]
